# Post-Cranial Skeletons of Hypothyroid Cretins Show a Similar Anatomical Mosaic as *Homo floresiensis*

**DOI:** 10.1371/journal.pone.0013018

**Published:** 2010-09-27

**Authors:** Charles Oxnard, Peter J. Obendorf, Ben J. Kefford

**Affiliations:** 1 School of Anatomy and Human Biology, and Forensic Science Centre, The University of Western Australia, Crawley, Western Australia, Australia; 2 School of Applied Sciences, RMIT University, Melbourne, Victoria, Australia; 3 Centre for Environmental Sustainability, Department of Environmental Sciences, University of Technology Sydney (UTS), Broadway, New South Wales, Australia; University of Utah, United States of America

## Abstract

Human remains, some as recent as 15 thousand years, from Liang Bua (LB) on the Indonesian island of Flores have been attributed to a new species, *Homo floresiensis*. The definition includes a mosaic of features, some like modern humans (hence derived: genus *Homo*), some like modern apes and australopithecines (hence primitive: not species *sapiens*), and some unique (hence new species: *floresiensis*). Conversely, because only modern humans (*H. sapiens*) are known in this region in the last 40 thousand years, these individuals have also been suggested to be genetic human dwarfs. Such dwarfs resemble small humans and do not show the mosaic combination of the most complete individuals, LB1 and LB6, so this idea has been largely dismissed. We have previously shown that some features of the cranium of hypothyroid cretins are like those of LB1. Here we examine cretin postcrania to see if they show anatomical mosaics like *H. floresiensis*. We find that hypothyroid cretins share at least 10 postcranial features with *Homo floresiensis* and unaffected humans not found in apes (or australopithecines when materials permit). They share with *H. floresiensis*, modern apes and australopithecines at least 11 postcranial features not found in unaffected humans. They share with *H. floresiensis*, at least 8 features not found in apes, australopithecines or unaffected humans. Sixteen features can be rendered metrically and multivariate analyses demonstrate that *H. floresiensis* co-locates with cretins, both being markedly separate from humans and chimpanzees (P<0.001: from analysis of similarity (ANOSIM) over all variables, ANOSIM, global R>0.999). We therefore conclude that LB1 and LB6, at least, are, most likely, endemic cretins from a population of unaffected *Homo sapiens*. This is consistent with recent hypothyroid endemic cretinism throughout Indonesia, including the nearby island of Bali.

## Introduction

Earlier studies of the bones from Liang Bua (LB) on the Indonesian island of Flores suggested that they could be: *Homo* but separate from modern humans, possibly showing island dwarfing, and possibly derived from *H. erectus* (hence the new species *H. floresiensis* [1], [2] ). Other workers implied that they could be modern *H. sapiens* but displaying a genetic dwarfism [3] such as Laron syndrome [4] but these hypotheses of genetic pathologies have been strongly challenged [5], [6]. Our earlier studies of the crania of hypothyroid endemic cretins, including morphometric analyses of quantitative data [7] suggested that they showed many features similar to those reported in the original diagnosis of the LB1 skull. New and more extensive studies of the upper and lower limbs [8], [9], [10] together with new studies of the cranium [6], [11] show that the Liang Bua remains display a curious mosaic of (a) derived characters as in modern humans, (b) primitive characters as in apes and australopithecines, and (c) unique characters. The derived features would suggest that these remains are of the genus *Homo*, the many primitive and unique features indicate that it is not *H. sapiens*, hence *H. floresiensis*.

The aim of this study is primarily to examine the postcranial skeletons of hypothyroid cretins (Table S1) in order to see if they have an anatomical mosaic like that of *H. floresiensis*. We use the terms ‘cretinism’ and ‘cretin’ for individuals born with congenital hypothyroidism and in whom the consequences of hypothyroidism are manifest in subsequent development (see, for example, a recent review by Chen and Hetzel [12], and the *Comprehensive Handbook of Iodine* [13] for examples of this usage). In current clinical practice these terms are not used, and are replaced by the term ‘congenital hypothyroidism’, because the condition is reversible by treatment with thyroxine, and to avoid any stigmatization of affected individuals. Hypothyroid cretins are characterized by a high level of hypothyroidism, and consequent extreme dwarfism, arising from a lack of a functioning thyroid gland. Congenital hypothyroidism may arise from genetic mutations, or may be caused by environmental factors, most important of which is maternal iodine deficiency. Hypothyroid endemic cretins, also termed myxoedematous endemic cretins or in the latest International Diseases Classification (ICD-10) as ‘congenital iodine-deficiency disease syndrome, myxoedematous type’ [14] are distinguished from neurological endemic cretins who may not have experienced post-natal hypothyroidism but whose central nervous systems are damaged *in utero* [12], [15]). In museum collections some individual skeletons of less reduced stature are labeled as cretins, and may indicate individuals of low intelligence who may have suffered hypothyroidism pre-natally but not through post-natal development, and do not show characteristic features of hypothyroid cretinism.

We find that hypothyroid endemic cretins display a similar post-cranial anatomical mosaic to that described for *H. floresiensis*. The features, in which the cretins are similar to the primitive characters of apes and australopithecines, are, in cretins, spurious similarities resulting from the growth disturbances. The features in which cretins are similar to unaffected humans are those not affected by the growth reductions and indicate that they are human. The features that are unique are variously due to differential aspects of the growth deficiencies in cretins. One example of such a differential is that lengths of bones are differently affected than widths and cortical thicknesses because of the differences between greater reductions of interstitial growth of epiphyseal cartilage (lengths) compared with lesser reductions of subperiosteal and subendosteal appositional addition or removal of bone (widths and cortical thicknesses).

Our results, therefore, are consistent with the cretinism hypothesis. The similarity of the cretin mosaics to those of LB1 and LB6 (where remains exist) imply that these individuals were affected by hypothyroidism or a hypothyroid-like growth disorder, and therefore suggest that it is unlikely that they represent a new species. Other individuals found at Liang Bua are represented by extremely limited material, including a single tooth. This makes it difficult to determine whether they are healthy individuals, children or pathological individuals. In the absence of primitive DNA at Liang Bua, there is no reason to attribute remains to a primitive species at a time well after *H. sapiens* has appeared in the region. Because, in LB1 and LB6, some of the cretin features are not yet found, not yet described, or hidden by damage, our results also supply a series of predictive tests that could be applied to further finds.

## Results

### The post-cranial anatomical mosaic

Some aspects of the anatomy of the *H. floresiensis* upper limb [16], [17] do seem to show primitive features not found in *H. sapiens*. Thus, the scapula has a rounded lower angle, the clavicle is short in relation to its width, humeral head torsion is 120 degrees, and there are high long bone width/length ratios. These are all primitive features found in chimpanzees, gorillas and australopithecines, but not in modern humans. We have found, however, that these are also features of some endemic hypothyroid cretins where slowed and pathological bone growth produces similar effects on the form of bone shafts and epiphyses, on bone lengths relative to widths, and on bone torsions (Tables 1, 2, 3, 4 and 5).

**Table 1:** Apparent primitive post-cranial features of cretins compared to these features in *Homo floresiensis* and unaffected *Homo sapiens*.

Feature	*H. sapiens* cretins (age 20–30 years)	*H. sapiens* cretins (age 67–80 years)	*H. flores-iensis*	Apes (and Australo-pithecines where available)	*H. sapiens* (healthy)	*H. sapiens* Micro-cephalic dwarf
Iliac blade orientation	**lateral**	**lateral**	**lateral**	**lateral**	antero-lateral	antero-lateral
Relative clavicle length	**39–42**	55–60*	37*	**37–42**	46–50	?
Long bone widths relative to lengths	**high**	**high**	**high^2^**	**high**	low	very low
Medial orientation of humeral head	**yes**	damaged by pathology*	missing	**yes**	no	no
Medial orientation of femoral head	**yes**	damaged by pathology*	**yes**	**yes**	no	no
Hand relative to both forearm and upper limb lengths	**large**	**large**	not available: parts missing	**yes**	small	v. small and delicate
Foot large relative to tibia	**76–80**	**77–84**	**76**	**80–92**	53	delicate: no measure
Foot large relative to lower limb	**39–43**	**35–39**	**44**	**46–47**	31	delicate: no measure

Bold type indicates features that are similar to cretins. Asterisks indicate differences between young adult and older cretins (which latter may have severe pathologies). Question marks indicate indeterminable features due unavailability (microcephalics). Additional data are provided in Tables 4 and 5, including all variables used in the PCA.

**Table 2:** Apparent derived (modern) post-cranial features of cretins compared to these features in *Homo floresiensis* and unaffected *Homo sapiens*.

Feature	*H. sapiens* cretins (age 20–30 years)	*H. sapiens* cretins (age 67–80 years)	*H. flores-iensis*	Apes (and Australo-pithecines where available)	*H. sapiens* (healthy)	*H. sapiens* Micro-cephalic dwarf
Clavicle Twist	**near zero**	**near zero**	**near zero**	high 50–57	**low: 5–15**	**low**
Gleno/axillary angle	**145–149**	134–135*	**135 est.**	112–119	**137**	**134–149**
Many muscle markings (compared with apes)	**very weak**	**very weak**	**very weak**	strong	**weak**	**very weak**
7 out of 8 human-like features of talus	**present**	**present**	**present**	absent	**present**	not known

Bold type indicates features that are similar to cretins. Asterisks indicate differences between young adult and older cretins (which latter may have severe pathologies). Additional data are provided in Tables 4 and 5, including all variables used in the PCA.

**Table 3:** Unique post-cranial features of cretins compared to these features in *Homo floresiensis* and unaffected *Homo sapiens*.

Feature	*H. sapiens* cretins (age 20–30 years)	*H. sapiens* cretins (age 67–80 years)	*H. flores- iensis*	Apes (and Australo-pithecines where available)	*H. sapiens* (healthy)	*H. sapiens* Micro-cephalic dwarf
Pelvic components	**unfused or partly fused**	**fused - with epiphyseal lines present**	**damaged? repaired? unfused?**	fused	fused	fused
Bone twists	**humerus,femur**	**femur**	**humerus**	no	no	?
Bone bends	**humerus, ulna**	**?**	**radius**	no	no	no
Spiral groove on humerus	**no**	**no**	**no**	yes	yes	?
Reduced trapezoid	**yes**	**no**	**yes (7.6–13.1)**	no	no	?
Intermembral index	**78–84 (3)**	**78–83 (3)**	**86 est.**	97–124	65–69	66 (1)
Sternebrae fusion	**separate, partial or complete**	**partial or complete**	**partial ?**	**separate or partial**	fused in adult	fused in adult
Lumbar vertebral height/depth	**low: 55%–62%**	**low: no value available**	**low 53%**	lowest value: 80%	lowest value: 70%	not available
Vertebral epiphyses	**some unfused**	**some unfused**	?	fused	fused	fused
Sacrum	**incompletely fused**	**incompletely fused**	?	fused	fused	fused
Ribs missing	**last 1 or 2**	**last 1 or 2**	?	normal	normal	normal

Bold type indicates features that are similar to cretins. Question marks indicate indeterminable features due to loss, damage or absence (*H. floresiensis*) or unavailability (microcephalics). Additional data are provided in Tables 4 and 5, including all variables used in the PCA.

**Table 4:** Assembled upper limb quantitative data and intermembral (IM) indices for cretins of three different age groups, *Homo floresiensis* and other species.

Species (data source)	Clavicle/humerus length ratio (×100) (clav/hum)	Gleno-axillary angle (deg.) (GA angle)	Humerus W/L ratio (×100) (hum W/L)	Radius W/L ratio (×100) (rad W/L)	Clavicle W/L ratio (×100) (clav W/L)	Twist of clavicle (deg.) (clav twist)	Upper/lower limb length ratio (×100) (IM index)
*Homo sapiens* (cretin 20–30 yrs) *(1)*	39, 41, 42	140, 145, 149	7.3, 7.1	7.8, 7.2	11, 7.9	5.0, 7.8, 10.8	76, 80
*Homo sapiens* (cretin 40 yrs) *(1)*	52	140	8.5	7.9	7.0	7.5	70
*Homo sapiens* (cretin 67–80 yrs) *(1)*	48, 52, 57	134,135,137	9.7, 8.8	7.7, 7.5	9.5, 9.0	4.0, 8.0, 10.0	71, 76, 80
*Homo floresiensis (2,3,4)*	37	138, 140, 142	7.2	6.9	9.3	3.5 est.	78
*Homo sapiens (5,6,8)*	mean 49, sd, 5.2	mean 137 (n = 66), sd. 3.6	6.5, 6.2, 6.0, 6.3, 6.0	5.9, 5.9, 6.2. 6.0, 6.0	7.6, 7.7, 7.7, 7.3, 7.3	mean 7.3, (n = 66), sd 5.6	64, 64, 65, 65, 71
*Australo-pithecus (5,6,8)*	No data	103	?	?	?	57	95–100 est.
*Pan troglo-dytes (5,6,7,8)*	37, 37, 41, 42	mean 113, (n = 57) sd, 6.8	10.3,10.6, 10.0, 10.1	5.9, 5.9, 5.8, 5.7	8.0, 8.2, 8.1, 8.3	mean 49, (n = 57), sd 9.8	105, 106, 109, 110 range of sample (n = 27), 101–114

Individual values are provided except where sample sizes are large enough to give mean and standard deviations. Abbreviation for each variable is provided for vector diagrams in Figures 2, 3. Data sourced from (1) this study, Basle Museum of Natural History, (2) ref. [9], (3) ref. [9], (4) ref. [10], (5) refs. [35]–[36] (6) ref. [37] (7) data gifted to CEO by A.H.Schultz, (8) materials at UWA and CEO.

**Table 5:** Assembled lower limb quantitative data for cretins of three different age groups, *Homo floresiensis* and other species.

Species (data source)	Foot/tibia length ratio (×100) (foot/tibia)	Foot/lower limb length ratio (×100) (foot/leg)	Navi-cular LL/ML (×100) (navic)	Meta-tarsal I/Meta-tarsal II length ratio (×100) (mtI/mtII)	Meta-tarsal I/meta-tarsal III length ratio (×100) (mtI/mtIII)	Proximal phalanx II/meta-tarsal II length ratio (×100) (ppII/mtII)	Proximal phalanx V/meta-tarsal V length ratio (×100) (ppV/mtV)	Femur W/L ratio (×100) (fem W/L)	Tibia W/L ratio (×100) (tibia W/L)
*Homo sapiens* (cretin 20–30 yrs) *(1)*	76, 80	3, 39	44, 54, 55	76, 78	81, 82,	41	36	7.2, 9.6	8.3, 7.6
*Homo sapiens* (cretin 40 yrs) *(1)*	No data	No data	No data	No data	No data	No data	No data	7.9, 7.1	7.1
*Homo sapiens* (cretin 67–80 yrs) *(1)*	76, 80	39, 43	52, 61, 64	80, 82	83, 88	41, 41	43, 45	8, 7.6, 7.6	8.3, 8.7
*Homo floresie-nsis (2,3,4)*	76	44	49, 50, (each side)	75	78	43	43	7.6	10.6
*Homo sapiens (5,6,8)*	50, 51, 51, 54, 55	27, 28, 30, 33, 34	85, 87, 87, 90, 99	28, 31, 33, 37, 40	80, 82, 85, 90, 95	31, 33, 36, 36, 40	28, 34, 34, 38, 40	6.7, 6.6, 5.4, 5.7, 5.5	5.6, 5.5, 6.0, 5.9, 5.9
*Australo-pithecus (5,6,8)*	No data	No data	51	No data	No data	No data	No data	?	?
*Pan troglo-dytes (5,6,7,8)*	76, 80, 86, 88	43, 44, 46, 48	40, 44, 45, 45	77, 78. 84, 88	67, 67, 68, 69	43, 44, 55, 60	42, 47, 54, 60	8.1, 8.9, 8.3, 8.5	7.3, 7.3, 7.4, 7.0

Individual values are provided except where sample sizes are large enough to give mean and standard deviations. Abbreviation for each variable is provided for vector diagrams in Figures 2,3. Data sourced from (1) this study, Basle Museum of Natural History, (2) ref. [9], (3) ref. [9], (4) ref. [10], (5) refs. [35]–[36] (6) ref. [37] (7) data gifted to CEO by A.H.Schultz, (8) materials at UWA and CEO.

In contrast, other features of upper limb anatomy of *H. floresiensis* are only found in humans, not in apes (or australopithecines where evidence is available), and might be described as derived. One example is the angle of the glenoid cavity on the scapula (gleno-axillary angle: 135 degrees in Liang Bua, 134–149 in cretins and 137–149 in normal humans, as contrasted with 112–119 in apes (and one australopithecine, see Figure S1). Another is the longitudinal twist of the clavicle (estimated as close to zero in Liang Bua and cretins, 5–15 degrees in normal humans, but 50–57 degrees in chimpanzees). In unaffected humans and cretins these features relate to weaker arms that hang mainly by the side and are not involved in body weight-bearing in contrast with their position in apes (and possibly australopithecines) where the shoulders are oriented more cranially and powerful upper limbs are involved in weight bearing locomotion.

The wrist bones also provide information. Tocheri et al. ([18]) describe the available wrist bones (attributed to LB1), particularly the trapezoid, as ape-like. This similarity is partly because the trapezoid is shown at a different magnification (125% larger) than the capitate. When the magnification is corrected then the trapezoid is too small to fit with the capitate. This is confirmed by the new published dorso-palmar measure of the LB1 trapezoid (7.6 mm) which is indeed small relative to the capitate (14.1 mm), ratio, 0.54, although described as complete [9]. A trapezoid like this, that is small relative to the capitate, is not found in *Ardipithecus ramidus* [19], or any ape or any normal human, but trapezoids are generally not described for early hominins [20]. Small trapezoids do, however, sometimes occur in adult human cretins because there may be delayed ossification of the ventral portion of the bone in younger cretins, or failure of fusion of the two parts of the bone with loss of the smaller ventral portion after death [7] [21]. A young adult cretin (Basle, specimen 84, male) shows exactly such an incomplete trapezoid lacking a ventral tip adjacent to a normal capitate. The trapezoid/capitate ratio of that cretin is 10.1/16.1 = 0.63 and similar ratios have been measured in other young adult cretins (our unpublished data). In the old cretins that we have examined, of age 67–80 years, in whom growth has continued long into adulthood, the ratio is, as in unaffected humans, 0.9–1.0. The small LB1 trapezoid, further, will not fit in the strongly curved dorsal curvature of the carpal row of a chimpanzee but does articulate well in the shallow dorsal curve of the human carpal tunnel (Figure S2). Radiography [22] and examination (BMNH 20 year old female) of young adult cretin wrists confirm that even in adulthood there may be fewer and smaller bony carpals as a result of delayed ossification. In conclusion, although the LB1 trapezoid is described as complete (in the sense that it does not show any evidence of a piece broken off it), it does appear to be incomplete. This is confirmed by the unusually small dorso-palmar measure of trapezoid compared to capitate and is similar to the small ratios found in younger adult cretins with incompletely ossified trapezoids.

The new studies of the lower limb [8], [10] also seem to show primitive features in *H. floresiensis*. These include a high upper limb/lower limb ratio (86% compared to as much as 97–124% in chimpanzees). This ratio is much less in unaffected humans (and microcephalic dwarfs) at 63–69%. Cretins, however, at 78–84%, are close to *H. floresiensis*.

*H. floresiensis* has a marked medial inclination of the femoral head upon the femoral neck as in apes and australopithecines. In unaffected humans the femoral heads and necks reach up towards the acetabulum and are strongly cranially oriented. In young adult cretins, however, femoral heads and necks are more medially inclined. In old cretins this medial orientation has degenerated into severe pathology with marked deformation of the femoral head. The medial orientation of young cretins is evident in apes (and australopithecines). Of course, in apes, it is associated with the hip joint being aligned for a degree of quadrupedal locomotion.

**Figure 1:**
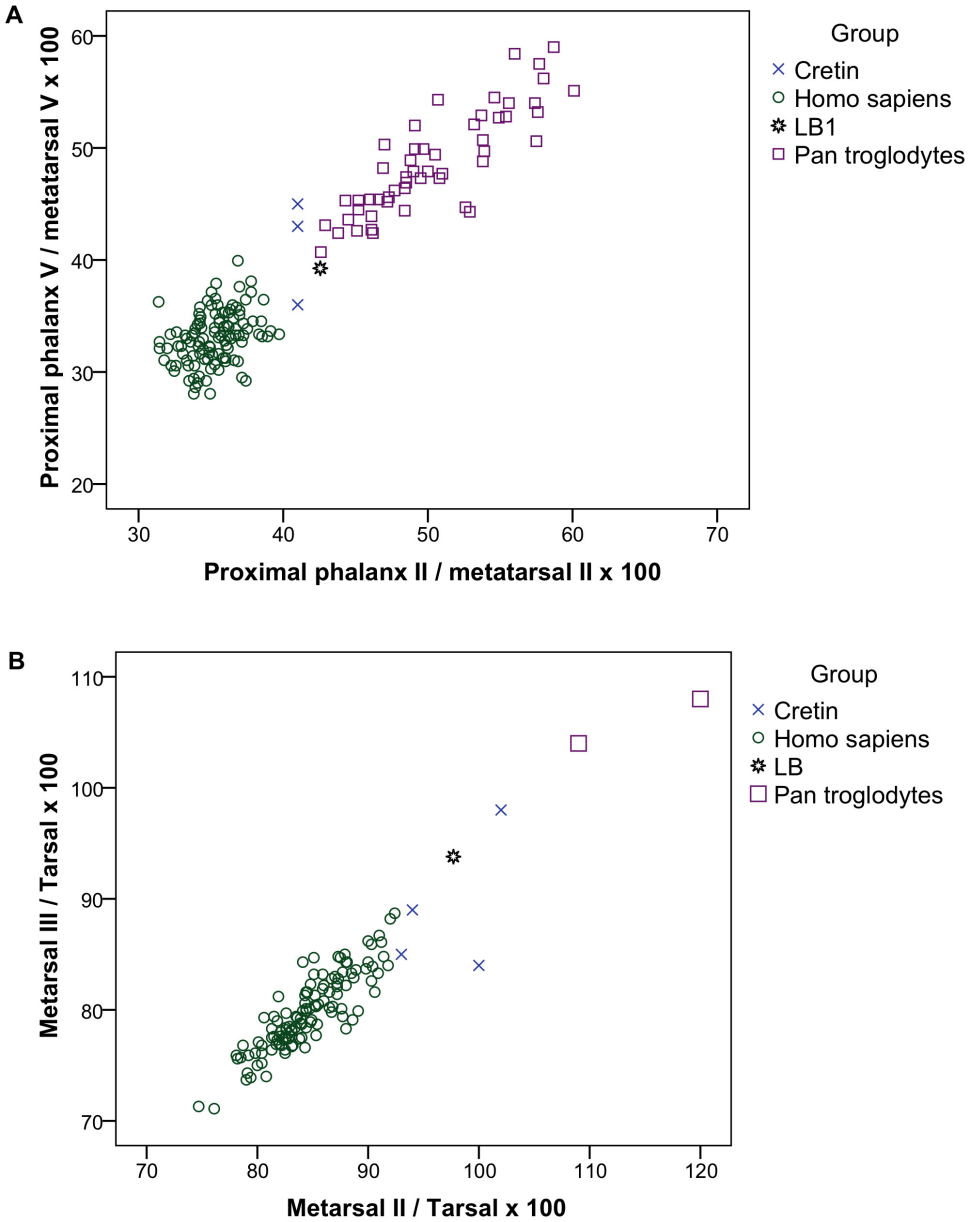
Intrinsic foot proportions in cretins and *Homo floresiensis* as published in [**10**] with data from 3 cretins (frame A) and four cretins and 2 *Pan troglodytes* (frame B) added. A. Length of proximal phalanx relative to length of metatarsal for digit II is plotted against similar ratio for digit V. After ref. [10] with cretin data added from Basle cretins. B. Ratio of metatarsal I length to tarsal length is plotted against the ratio of metatarsal II length to tarsal length. After ref. [10] with cretin data from Basle and two chimpanzees (*Pan troglodytes*) added, and with the same definition of tarsal length, that is from the back of the talus forward not, as usually, from the posterior limit of the calcaneus.

The foot of LB1 has a series of features that are of interest. Thus it has a long foot relative to leg length (76%) and lower limb length (44%) and in these measures it is similar to apes (80–92% and 46–47%, respectively) but completely different from unaffected humans (53% and 31%, respectively). Though cretins have absolutely small feet, they have even shorter limbs so that the ratios of foot length to leg length and lower limb length have values (76–84% and 35–43%) similar to apes. Further, LB1 has ape-like metatarsal and phalangeal ratios, and ape-like morphology of individual tarsal bones. Again, these same features are also found in cretins (Tables 1, 2). We have found that addition of cretin data to the published plot of phalangeal/metatarsal ratios shows human cretins locating close to *H. floresiensis* (Figure 1A). Likewise, in another published plot of metatarsal/tarsal ratios, LB1 is located outside of modern humans and towards chimpanzees but, when cretins are added, they, too, are located near LB1 (Figure 1B). Further to the foot data, although it is noted [10] that the talus has one feature in which it is like apes (reduced head torsion at 2–4 standard deviation units below the human mean), 7 other features of the talus are reported as human-like to such a degree that “LB1/15 is similar to modern humans in overall morphology” [10].

Some other features of the *H. floresiensis* limb bones are uniquely different from any other species, extant or fossil, ape or human. For example, there is “no detectable spiral/radial groove on the posterior aspect of the shaft” of the humerus [9]. This groove is an obvious feature of humans and is especially strong in apes. It is, however, (together with many other muscular markings throughout the skeleton) very weak or absent in cretins. Again, though the bend in the radius (LB1/62) is described as an old healed fracture the overall form of the figured radiograph shows no evidence of a remodelled callus [9] and the photograph of the radius (fitting the radiograph) is similar to the bends that can also be found in individual bones of cretins (e.g. humerus and ulna, Figure S3). Finally, though not mentioned in the above references, the ratio of the height of a lumbar vertebral body of LB compared with its dorso-ventral dimension (at 53% measured on a photograph) is totally different from unaffected humans. The lowest value in unaffected humans is 70.5% in the 5^th^ lumbar vertebra and the lowest value in chimpanzees is even higher). The values for four lumbar vertebrae in a cretin range from 55% to 62%, very similar to Liang Bua. The reduced ratio in cretins is because the vertebral epiphyses remain cartilage thus reducing the vertical dimension of the body.

In summary (refer Tables 1, 2 and 3), the limbs of cretins show post-cranial anatomical mosaics similar to *H. floresiensis*.

### Multivariate Analysis

In order to summarize the data and show the location of cretins in relation to chimpanzees, unaffected humans and *H. floresiensis*, we have undertaken principal components analyses (PCA) using the available metrical variables (Tables 4, 5). Because there were missing values for some individual cretins, it was possible to carry out the analyses either: on a reduced number of variables with larger numbers of specimens (but this might be seen as cherry picking variables) or the full number of variables with reduced numbers of specimens (which involves having fewer specimens than variables). The maximum number of available variables, 16, was larger than the maximum number of individuals with a full set of values for those variables, 13. No particular problem arises from this in PCA when the goal is to compare specimens and not to test statistical significance (unlike, for example, discriminant analysis or canonical correlation) because the covariance matrix can be singular, leading to some eigenvalues being zero and ignored (see Rencher [23] for a discussion of this). In order to represent all data, and to check the effect of decreased variables in relation to more individuals, we have repeated the PCAs with reduced sets of variables and increased numbers of individuals thus providing analyses with more specimens than variables.

**Figure 2:**
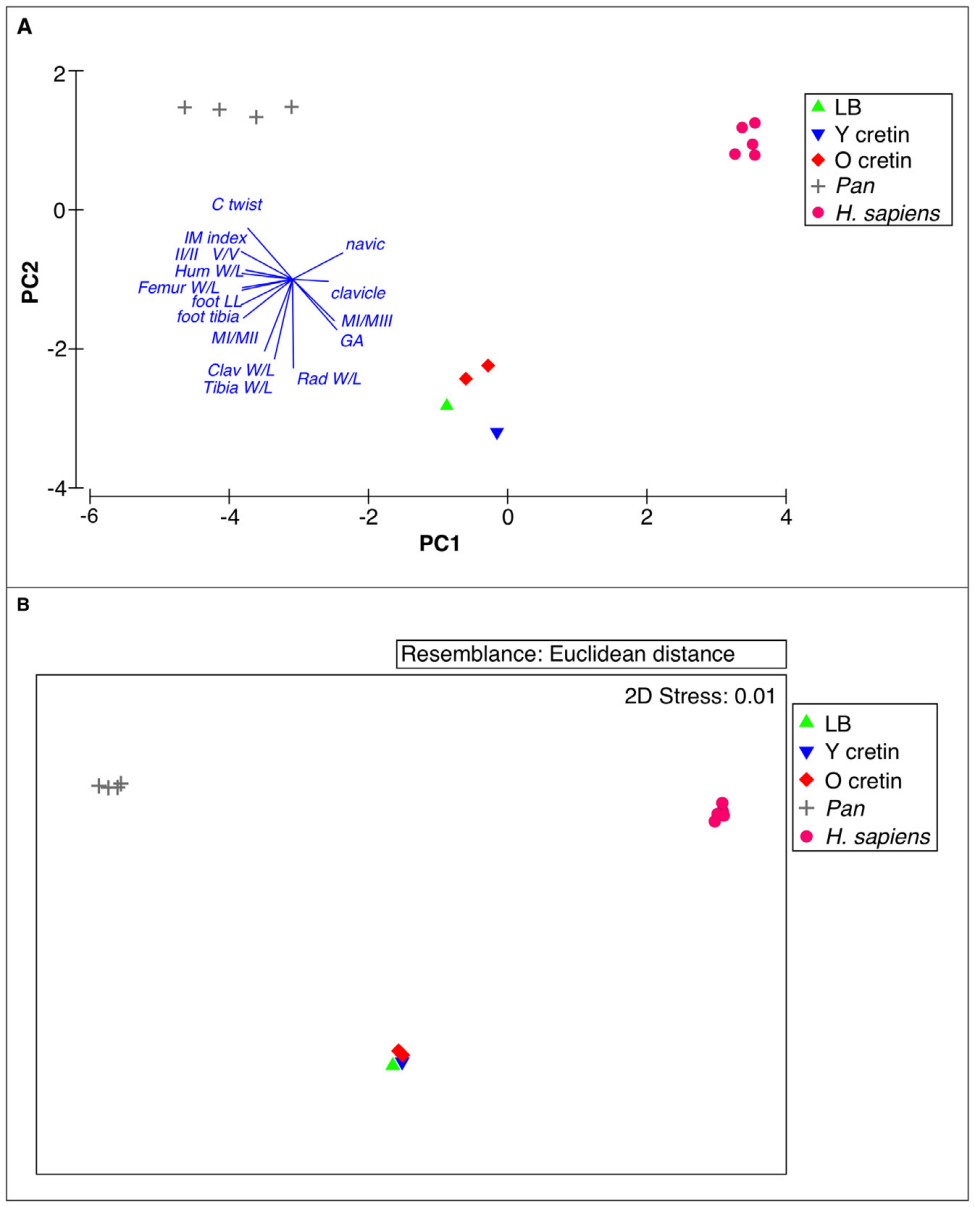
Multivariate analyses of quantitative features of *Homo floresiensis* in relation to cretins, unaffected humans and chimpanzees. Individuals are represented for each specimen by the coloured symbols above: *H. floresiensis*, young adult cretins, older cretins, *H. sapiens*, and *P. troglodytes*. Vectors are shown for each variable, abbreviated as in Tables 4, 5. The direction of each vector indicates the association with each axis and the length indicates the strength of the association. A. Principal Components Analysis (PCA) of all variables (16, from Tables 4, 5), 13 individuals, including 1 young adult cretin, 2 older cretins. PC1 and PC2 together explain 85.8% of variability. Two pairs of nearly coincident vectors (ppII/mtII, ppV/mtV and femur W/L, foot/leg) have similar directions and lengths. Three groups are clearly defined in 2 dimensions and confirmed over all dimensions (ANOSIM global R>0.999, P<.001). The group of LB and cretins is separate from *H. sapiens* (ANOSIM R>0.999, P<0.01) and *Pan* (R>0.999, P = 0.03). B. Multi-Dimensional Scaling (MDS) with individuals and variables as in a). Two Dimensional stress was 0.01 indicating that the plotted values give a very good indication of the rank Euclidean distance between individuals.

**Figure 3:**
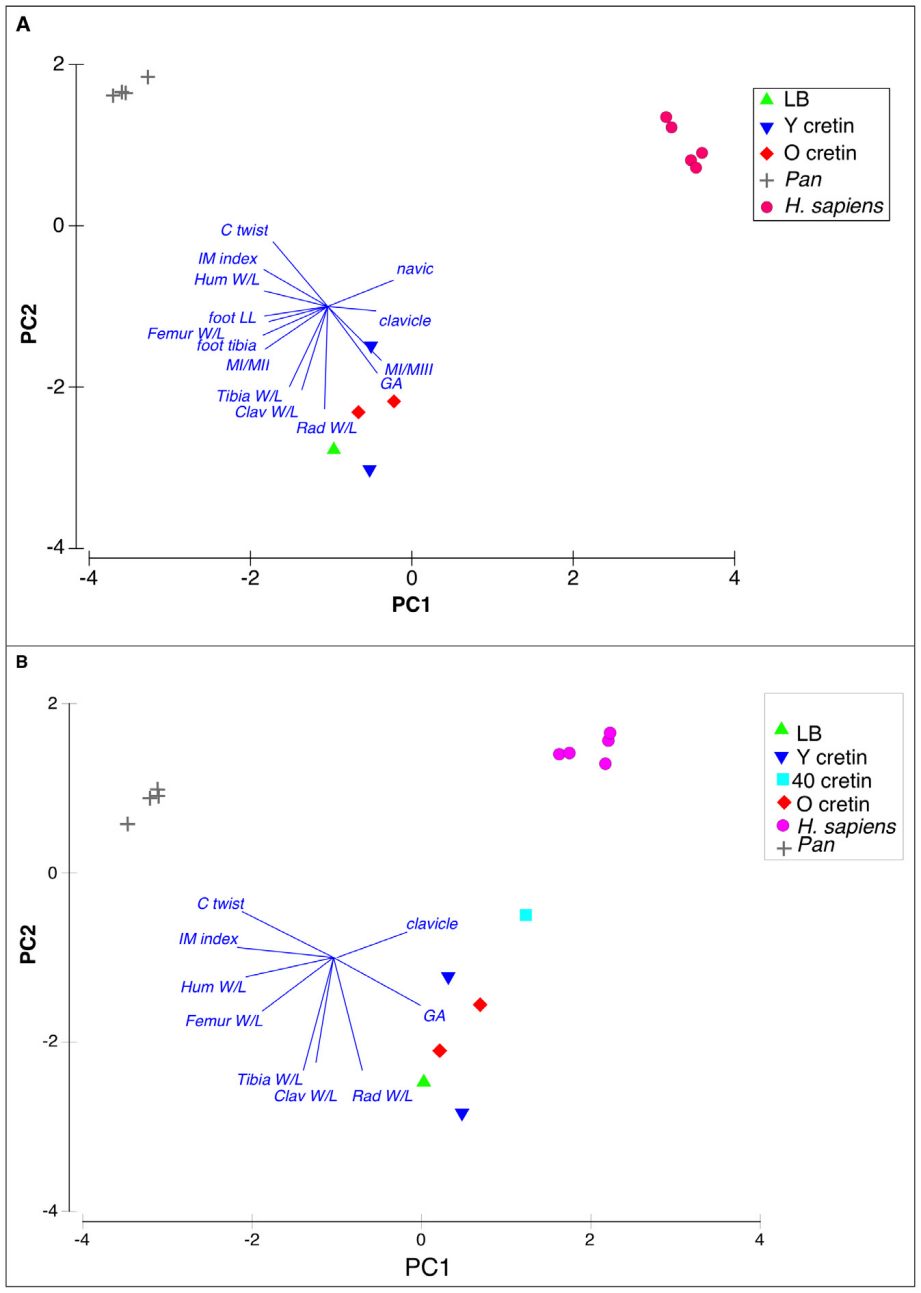
Additional multivariate analyses of quantitative features of *Homo floresiensis* in relation to cretins, unaffected humans and chimpanzees. A. PCA using 14 variables and 14 individuals, as in a) but with 2 young adult cretins. PC1 and PC2 together explain 85.3% of variability. Three groups are clearly defined (ANOSIM global R>0.999, P<.001). The group of LB and cretins is separate from *H. sapiens* (ANOSIM R>0.999, P<0.01) and *Pan* (ANOSIM R>0.999, P<0.01). B. PCA using 9 variables, and 15 individuals, as in b) with additional 40 year old cretin. PC1 and PC2 together explain 79.9% of variability. ANOSIM global R = 0.949, P<.001. Group of LB and cretins is separate from *H. sapiens* (ANOSIM R = 0.843, P<0.01) and *Pan* (ANOSIM R>0.999, P<0.01).

In each case (Figure 2 A, Figure 3 A, B), the PCAs show that the hypothyroid cretins locate close to *H. floresiensis* (composite of LB1 and LB6); both are far distantly located from humans and chimpanzees. The first principal component (PC1) clearly separates, without overlap, three groups: all humans at one extreme, all chimpanzees at the other, and all cretins together with *H. floresiensis* intermediate. The second, PC2, provides further marked separation of cretins and *H. floresiensis* from both humans and chimpanzees so that the true relationship of the cretin/*H. floresiensis* group to humans and chimpanzees is triangular not simply intermediate. As expected from these clear separations in the PCAs, Analysis of Similarity (ANOSIM) confirms that the group of *H. floresiensis* and cretins is separate from the groups of humans and of chimpanzees at a very high level of confidence in each case (global ANOSIM R>0.999, P<0.001).

Non-metric Multi-Dimensional Scaling (MDS), a method with a different statistical base to PCA, is shown in Figure 2 B and this confirms the close grouping of *H. floresiensis* with the hypothyroid cretins, and the clear separation of this group from both modern humans and from chimpanzees.

## Discussion

It is clear that human cretins share some post-cranial features with *Pan*, *Gorilla*, and even *Pongo* among living species, and australopithecines, (and even, in some few available features, *H. erectus*) among the fossils. They share other features with unaffected humans where these are different from apes and fossil hominins (Tables 1, 2 and 3). Cretins have yet other features that are not found in adults of apes or modern humans (i.e. they appear unique). In cretins these features are mainly because they sometimes appear absent due to being still cartilage well into adulthood, whilst in adults of all unaffected species (humans and apes alike) they are present as bone. These cretin mosaics (slightly different from one cretin to another) should be seen alongside the mosaic of primitive (ape-like), derived (human-like) and unique morphologies in *H. floresiensis* [8], [9], [16] which are, thus, incorrectly described as mosaics that are a “combination … never found in … pathological modern humans” e.g. [9].

The PCA and MDS show a very clear and robust result, confirmed by ANOSIM, which, for these variables, the *H. floresiensis* post-cranial mosaic replicates those of hypothyroid cretins. Taken with the similar result previously reported for their cranial morphologies [7], the conclusion is inescapable that the *H. floresiensis* individuals show general anatomical features similar to those caused by congenital hypothyroidism.

The best estimate dates for LB6 (15 ka B.P.) and LB1 (18 ka B.P.) are much later than the 42 ka B.P. presence of modern humans on the then almost contiguous island of Timor [24]. This renders the assignment of these remains to a species other than *H. sapiens* very unlikely. An exposed sediment layer containing stone artefacts on Flores has been dated to 1.0 ma, implying that another hominin species had reached Flores [25], and if this is confirmed, it is possible that the LB remains represent descendants of that species. If this is the case, and this would need to be confirmed by ancient DNA to be certain, then our data would imply that at least some individuals of that descendant species were affected by hypothyroidism.

It is also remotely possible that these individuals are rare sporadic (that is, genetic) cretins. However, as at least two individuals with some of these features are present at Liang Bua and separated by several thousand years, it seems more likely that they are myxoedematous (hypothyroid) endemic cretins. Endemic cretinism has been observed at high prevalence (up to about 10% of individuals affected) in some small human populations and has especially been common in this region in recent times.

Hypothyroid endemic cretinism arises when environmental factors, including iodine deficiency, selenium deficiency, and raised serum thiocyanate, result in the destruction of thyroid cells *in utero*. Evidence for these environmental factors obtaining in a population of inland hunter-gatherers on Flores has been presented [7]. One published criticism of the cretinism hypothesis is an assertion that extreme iodine deficiency was unlikely on such a small island as Flores because of presumed access to marine foods [26], but we now note that high rates of visible goitre and hypothyroid cretinism have been widely observed on the nearby, even smaller and geologically similar island of Bali [27], and large numbers of confirmed cases are reported on Java, Sumatra and Borneo [7]. As soil iodine levels can fall markedly even as little as 18 km from the coast [28], and if the other factors (thiocyanate excess, and selenium deficiency) are present, endemic cretinism may be found in populations at this distance from a marine coast.

There are also cranial features that should be considered. Thus, the sella turcica, enlarged in some cretins, is described as not enlarged (about 8 mm) in LB1 [5]. In our cretin skulls (Table S1) the entrance to the sella turcica (Figure S4, Figure S5) ranges from about 8 mm (with a large ballooned space about 13 mm long inside the bone) to about 13 mm (with a smaller internal space of only 8 mm). This is consistent with the variability described in Chinese hypothyroid cretins [29] whose sella turcica mean size of 14 mm had a standard deviation 3.1 (n = 58). This implies that many of the Chinese cretins are as small as 8 mm - 2 standard deviations less than the mean. In the LB1 endocast the concave sella appears as a raised convexity which we estimate as about 8–9 mm long. The projecting openings of the sella appear as two deep pits and these are about 12–13 mm apart. Although the detailed morphology and size remain undescribed, and the extent of damage is unclear, the LB1 sella is entirely compatible with the variations to be found in hypothyroid cretinism.

There is also the question of brain size. At 417 ml the reconstructed endocranial volume of LB1 is totally excluded from being a normal human but is characteristic of present day apes and australopithecines (and also of three of the smallest of the genetic microcephalics [3]). Cranial volumes in cretins, though indeed small compared with unaffected humans, are bigger than this, ranging from 670–1,150 ml (n = 5). This range is, however, for cretins of European ethnic origin (mean of 1,300 ml in unaffected individuals). A parent population that was much smaller than European would give rise to proportionally smaller cretins. Thus, normal South East Asian pygmoid crania of 800–1000 ml have been recorded (refs in [7]) and estimated [30]). On this basis, cretins from such populations could have brain sizes as small as 400–500 ml, based on scaling of height and brain size found among European endemic cretins [7].

A recent review has called for evidence that falsifies the new species hypothesis [31]. The best test of an evolutionary diagnosis for the LB remains would be determination of mitochondrial DNA sequences of these individuals. The demonstration of ancient DNA would falsify all hypotheses that these are pathological *H. sapiens*. The demonstration of *H. sapiens* DNA has the potential to falsify the new species hypothesis. “So far, however, only modern human DNA sequences have been found, consistent with handling of the specimens by multiple individuals prior to sampling” (personal communication, Alan Cooper). These unpublished results may, however, also be consistent with the presence of genuine, non-contaminant, modern human (hence *H. sapiens*) sequences, as the sequences of indigenous modern human Floresians of 15–18 ka BP would be largely indistinguishable from those of many present day inhabitants of Flores and other Indonesian islands. Considering the scientific importance of this question, and the availability of *in situ* teeth of two individuals, it is surprising to us that the status of the DNA has not been resolved.

Lacking such a conclusive test, we suggest that cretin anatomy provides many predictions for further testing the status of *H. floresiensis*.

We predict that if LB1 were a young adult cretin then the sternum would show either evidence of recent fusion of sternebrae or even some separate sternebrae. The sternum has not been described but a poor photograph suggests that fused but distinguishable sternebrae may be present [32]. Delayed ossification of the sternum of adult cretins (age 17 to 30) means that some show separate sternebrae (as in children) and others show fused but distinguishable sternebrae. It is only in some of the very oldest cretins (circa 70 years) that, like most unaffected humans, the sternum shows no sign of individual sternebrae. Of course, in cretins this feature mimics the primitive state, separate sternebrae or fused but distinguishable sternebrae being characteristic of adult apes (Figure S6).

We predict that some of the epiphyses in the Liang Bua materials will be confirmed as missing, unfused, or only partially fused (as in adult cretins but not adult apes or humans). This is so far not certain because many of these regions are reported as damaged. Searches for separated epiphyses and small missing pieces might be useful.

We predict that further evidence of the growth deficit of cretins may be found in the LB carpals and tarsals. As described above, one wrist bone, the trapezoid, is small compared with its neighbor; it is evident that another, the lunate, is incomplete; one tarsal bone, the navicular is distorted. These three features are common in cretins. Are such reductions in LB due to damage, loss or deficit?

We likewise predict that if the hands of LB1 are found, they will be large relative to upper limb length, because, with the relatively large feet of cretins go relatively large hands. These, again, resemble the primitive state - apes also have relatively large feet and hands, but in different locomotor contexts. So far not enough of the hands of LB1 are known to predict their size relative to the other upper limb bones.

We predict that more careful examination of both LB patellae may indicate that their curious shape, with two lunar shaped deficits on each supero-lateral aspect, is very similar to that of patellae with unfused epiphyses found both in unaffected human children and in growth-delayed 20–30 year old adult cretins (Figure S7).

We predict that if more vertebral bodies are found for LB1 they will appear to be cranio-caudally flattened. Unaffected humans have cranio-caudal/dorso-ventral vertebral body ratios ranging from a maximum of 105% for the first thoracic to a minimum of 70.5% for the last lumbar vertebra. Chimpanzees have even larger ratios. One cretin that we can measure has, in contrast, ratios of 55%–62% in each of four lumbar vertebrae. This reduction is partly because, even in some younger adults, the vertebral epiphyses remain cartilage and are not preserved thus reducing the vertical dimension. A single lumbar vertebral body is known for LB and it shares with the cretins a ratio of 53% [33].

We predict that examination of other vertebrae and sacra (if found LB1) will show evidence of partially or fully open sutures. This is certainly true of some cretin vertebrae and sacra (especially younger adults, Figure S8).

We predict the possibility that the separation of some parts of the *H. floresiensis* pelvis and cranium could be due to non-fusion, but testing of this will be difficult due to missing parts, taphonomic damage and laboratory reconstruction. Certainly some younger adult cretins have separate innominate components, and partially or even completely open spheno-occipital synchondroses, and inter-sphenoid and inter-occipital sutures.

In conclusion, as a result of the changes due to hypothyroidism, individual cretins show, throughout the entire skeleton, mixtures of features, some like unaffected humans, some like apes, and some unique, that, despite assertions to the contrary, are rather like the character mosaic described for the Liang Bua remains. Therefore these remains are most likely to be endemic cretins of species *H. sapiens*, a species known to be present in the region from at least 42 ka B.P. and not a new species. That they may be endemic cretins of another hominin species is acknowledged but is a more remote possibility.

## Materials and Methods

Mounted skeletons of hypothyroid cretins, many with autopsy data, were examined at the Basle Museum of Natural History, as well as other specimens (Table S1). Some observations and data on cretins are taken from the literature as indicated. Human and ape data, where not included in plots from the literature, were taken from specimens available to CEO. Most LB observations and measurements are as published, but some were taken from enlargements of published images.

Principal components analysis (PCA) was correlation based, and non-metric multi-dimensional scaling (MDS) and analysis of similarity (ANOSIM) were based on Euclidean distances. These analyses were conducted using the software Primer 6 [34]. For the PCA, all variables were first normalized by subtraction of mean and division by standard deviation. P values in the ANOSIM analysis were calculated for 999 random changes of labeling of groups.

## Supporting Information

Table S1 **Skeletons and isolated skulls examined and measured.**
(0.05 MB DOC)

Figure S1 **Gleno-axillary angles of scapulae of hypothyroid cretin, Homo floresiensis and other species.** The left two frames show the scapulae of an ape and an australopithecine (the specimen originally known as *Plesianthropus transvaalensis* and figured by John Robinson before the specimen became damaged: ref. [38]). These have gleno-axillary angles of about 90 degrees. The right three frames show the scapulae of LB 6/4, a cretin (Basle specimen 66, aged 28), and a modern human, and these all have a much larger angle. From ref. [39].
(0.09 MB DOC)

Figure S2 **Upper frames: carpal rows in chimpanzee and human with capitate and trapezoid shaded; lower frames: articulation of capitate and trapezoid from** ***H. floresiensis*** **showing dorsal carpal curvature that is produced.** The lower left figure shows that the *H. floresiensis* (LB1) trapezoid (dorso-palmar length 7.6 mm) is too small to articulate fully with the capitate (dorso-palmar length 14.1 mm) and that its articulation produces a flat dorsal carpal surface unlike that in the chimpanzee above it. The lower right figure shows that the LB1 trapezoid does fit into the shallow carpal tunnel of humans with the proposed deficit (arising from incomplete ossification due to delayed development) of the trapezoid shown as dotted lines. From ref. [39].
(0.08 MB DOC)

Figure S3 **Torsions and bends of long bones in hypothyroid cretins.** Torsions of humerus (left) and femur (middle) in cretins (Basle # 65 and # 64) with photograph of an adult cretin (right) showing such torsions in the thigh in the living (from image published in *Der Endemische Kretinismus*, 1936 [40], attributed to Dr L. D. Eerland, see reference [41] for context). Note also the relatively large hands and feet in the cretin individual. Bends in the humerus and ulna in cretins shown in reference [42] can be compared with the bend of radius in *Homo floresiensis* (LB 6/2).
(0.08 MB DOC)

Figure S4 **Extremes of sella turcica in cretins.** a) Sagittal section showing narrow opening and ballooned interior of Basle specimen 85. b) Dorsal view showing wide opening and reduced interior of Basle specimen 65 (dashed arrows of this specimen indicate lost (unossified) epiphyses of tips of clinoid processes).
(0.47 MB DOC)

Figure S5 **Variations in pituitary fossa form and measurement in normal and cretin humans.** The left diagram shows a saggital section of the region of the pituitary fossa in a non-affected human. The mouth and pit of the pituitary fossa are approximately the same width. The right diagrams show variations in pituitary fossa forms in cretins. The upper one shows a ballooned internal pituitary fossa (containing the enlarged gland) with a normal mouth. The lower one shows a small pituitary fossa with a wide mouth allowing for a pituitary gland to extend well beyond the confines of the bony fossa. From reference [39].
(0.10 MB DOC)

Figure S6 **Sternal form in apes, humans and human cretins.** Left side, line drawings show typical sternal form in orang utans (upper row), chimpanzees (second row), gorillas (third row) and humans (final row). The apes show mostly separate sternebrae, but with some partially fused sternebrae, and one with evidence of vertical fusion of the two sternal halves. Middle photographs, upper and lower, show *en face* sternums with some separate, some partially fused and some completely fused sternebrae in adult (17–30 year old) cretins. These can be compared to images of saggital sections of sternums with completely separate sternebrae in immature cretins in reference [42]. Right side, three photographs show increased fusion of sternebrae in cretins including one cretin in which the sternum is entirely like a normal human, and one cretin showing a vertical line of fusion (perhaps from the original two halves of the developing sternum) in three cretins aged 67–80 years. *En face* photographs from Basle specimens, 64, 65, 66, 84 and 85.
(0.09 MB DOC)

Figure S7 **Patella of LB1 compared to developing unaffected humans and young adult cretin.** The right patella of LB1 is shown in anterior view (a) and posterior view (d). Radiographic views of right patellae of a 15 year old (b) and of 8 year old humans (e). Anterior views of right patellae of 28 year old cretin, Basle specimen 66 (c) and 40 year old cretin, Basle specimen 578, f). Short white arrows show limits of concave facets for articulation of epiphyses (when present); red arrows show line of fusion of epiphyses when present.
(0.73 MB DOC)

Figure S8 **Sacra of hypothyroid cretin compared to unaffected human and chimpanzee.** Non-fusion of the many parts of the cretin sacrum (middle) is clear. Normal human (left) and chimpanzee (right) show the fused adult state.
(0.08 MB DOC)
