# Supplementary material for: Post-Cranial Skeletons of Hypothyroid Cretins Show a Similar Anatomical Mosaic as Homo floresiensis
Source: PLoS One. 2010 Sep 27;5(9):e13018. doi: 10.1371/journal.pone.0013018 (PMC2946357; doi:10.1371/journal.pone.0013018)
Supplement: Table S1 — Skeletons and isolated skulls examined and measured. (0.05 MB DOC) [file pone.0013018.s001.doc]

| Catalogue number | Sex, age and condition | |
| --- | --- | --- |
| # 1633: Natural History Museum, Basle | Male, 17 years, partial cretin skeleton | |
| # 84: Natural History Museum, Basle | Male, approx. 20 years, cretin skeleton | |
| # 66: Natural History Museum, Basle | | Female, 28 years, cretin skeleton |
| # 578: Natural History Museum, Basle | Female, 40 years, partial cretin skeleton | |
| # 65: Natural History Museum, Basle | Female, 67 years, cretin skeleton | |
| # 64: Natural History Museum, Basle | Female, 75 Years, cretin skeleton | |
| # 85: Natural History Museum, Basle | Male, 80 years, cretin skeleton | |
| Royal College of Surgeons of England, London | Adult, (half sternum only) cretin | |
| British Museum of Natural History, London BMNH 1949.12.7.16 | Female, 20 years, cretin skeleton | |
