# Supplementary figures and images for: Post-Cranial Skeletons of Hypothyroid Cretins Show a Similar Anatomical Mosaic as Homo floresiensis
Source: PLoS One. 2010 Sep 27;5(9):e13018. doi: 10.1371/journal.pone.0013018 (PMC2946357; doi:10.1371/journal.pone.0013018)

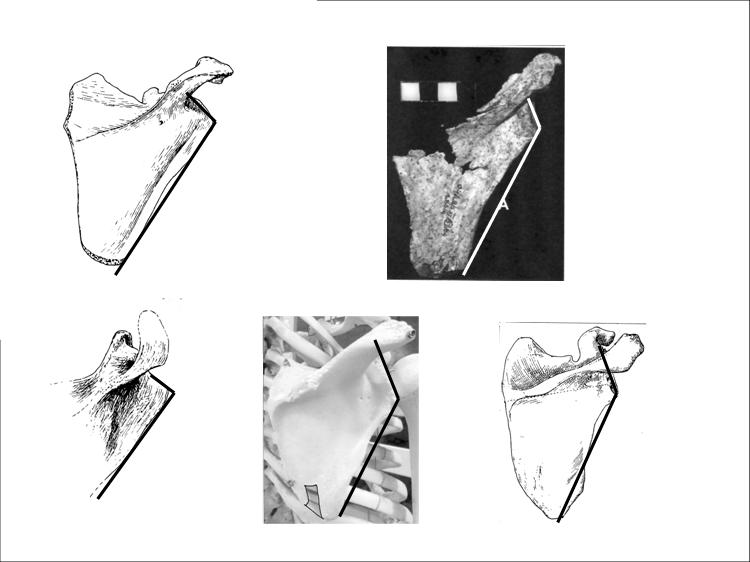

Supplement: Figure S1 — Gleno-axillary angles of scapulae of hypothyroid cretin, Homo floresiensis and other species. The left two frames show the scapulae of an ape and an australopithecine (the specimen originally known as Plesianthropus transvaalensis and figured by John Robinson before the specimen became damaged: ref. [38]). These have gleno-axillary angles of about 90 degrees. The right three frames show the scapulae of LB 6/4, a cretin (Basle specimen 66, aged 28), and a modern human, and these all have a much larger angle. From ref. [39]. (0.09 MB DOC) [file pone.0013018.s002.doc]

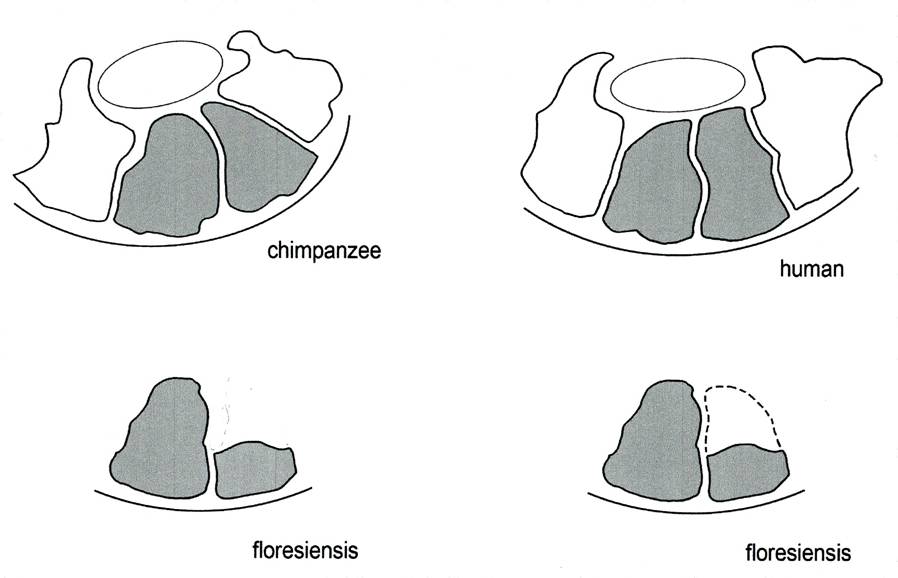

Supplement: Figure S2 — Upper frames: carpal rows in chimpanzee and human with capitate and trapezoid shaded; lower frames: articulation of capitate and trapezoid from H. floresiensis showing dorsal carpal curvature that is produced. The lower left figure shows that the H. floresiensis (LB1) trapezoid (dorso-palmar length 7.6 mm) is too small to articulate fully with the capitate (dorso-palmar length 14.1 mm) and that its articulation produces a flat dorsal carpal surface unlike that in the chimpanzee above it. The lower right figure shows that the LB1 trapezoid does fit into the shallow carpal tunnel of humans with the proposed deficit (arising from incomplete ossification due to delayed development) of the trapezoid shown as dotted lines. From ref. [39]. (0.08 MB DOC) [file pone.0013018.s003.doc]

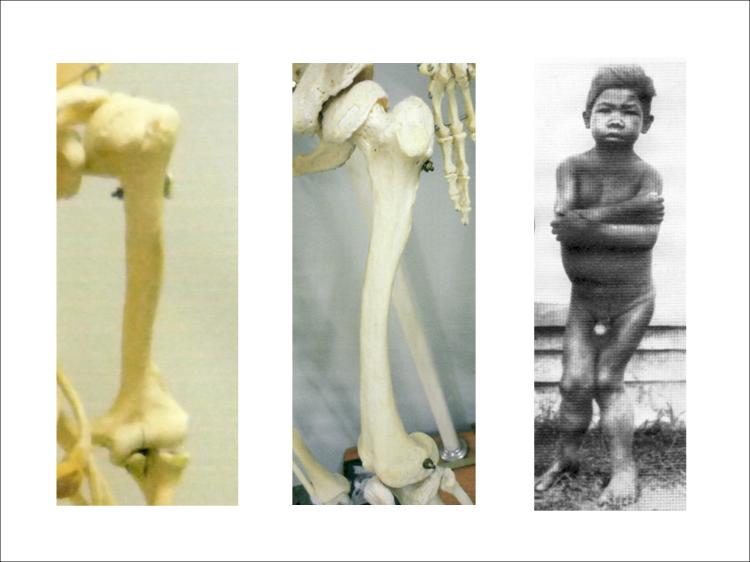

Supplement: Figure S3 — Torsions and bends of long bones in hypothyroid cretins. Torsions of humerus (left) and femur (middle) in cretins (Basle # 65 and # 64) with photograph of an adult cretin (right) showing such torsions in the thigh in the living (from image published in Der Endemische Kretinismus, 1936 [40], attributed to Dr L. D. Eerland, see reference [41] for context). Note also the relatively large hands and feet in the cretin individual. Bends in the humerus and ulna in cretins shown in reference [42] can be compared with the bend of radius in Homo floresiensis (LB 6/2). (0.08 MB DOC) [file pone.0013018.s004.doc]

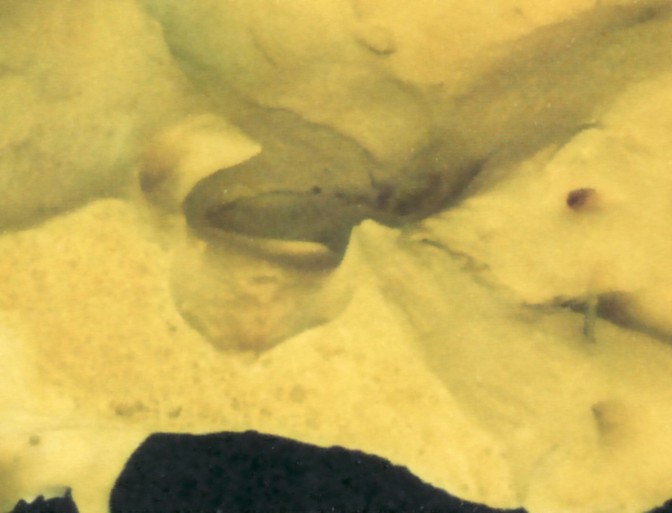

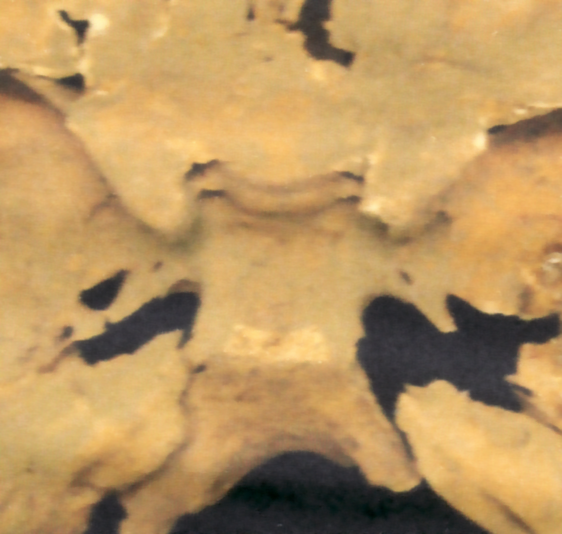


a

b

Supplement: Figure S4 — Extremes of sella turcica in cretins. a) Sagittal section showing narrow opening and ballooned interior of Basle specimen 85. b) Dorsal view showing wide opening and reduced interior of Basle specimen 65 (dashed arrows of this specimen indicate lost (unossified) epiphyses of tips of clinoid processes). (0.47 MB DOC) [file pone.0013018.s005.doc]

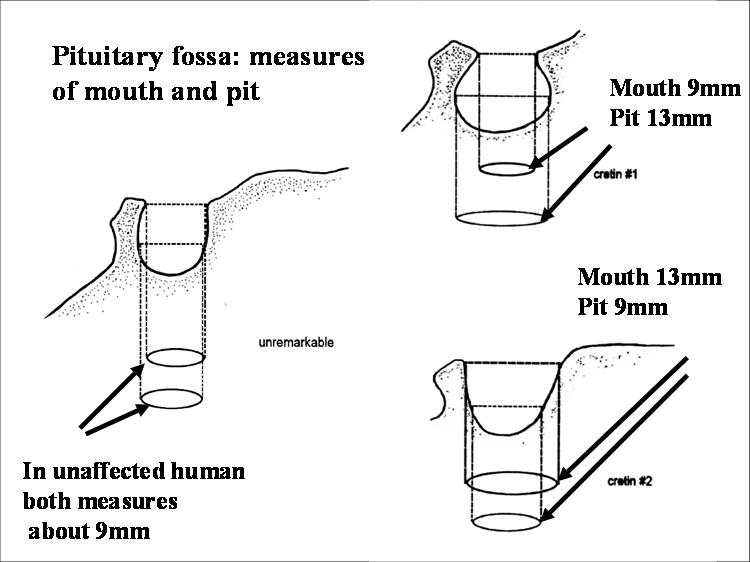

Supplement: Figure S5 — Variations in pituitary fossa form and measurement in normal and cretin humans. The left diagram shows a saggital section of the region of the pituitary fossa in a non-affected human. The mouth and pit of the pituitary fossa are approximately the same width. The right diagrams show variations in pituitary fossa forms in cretins. The upper one shows a ballooned internal pituitary fossa (containing the enlarged gland) with a normal mouth. The lower one shows a small pituitary fossa with a wide mouth allowing for a pituitary gland to extend well beyond the confines of the bony fossa. From reference [39]. (0.10 MB DOC) [file pone.0013018.s006.doc]

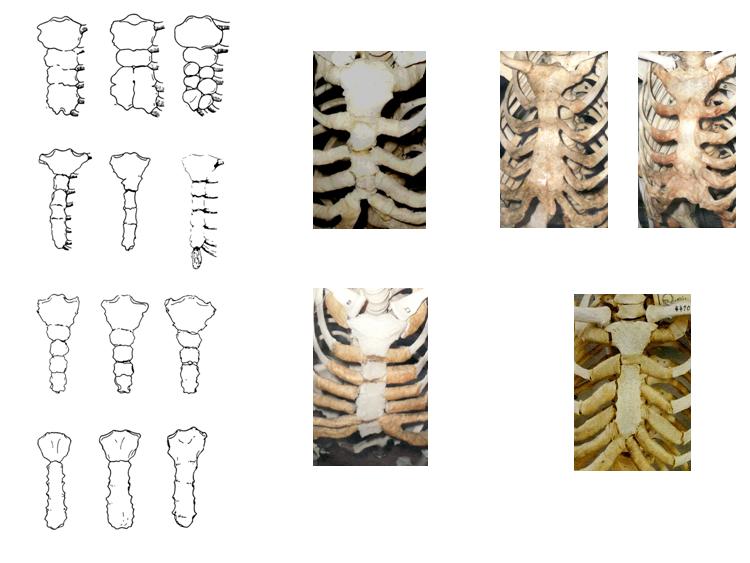

Supplement: Figure S6 — Sternal form in apes, humans and human cretins. Left side, line drawings show typical sternal form in orang utans (upper row), chimpanzees (second row), gorillas (third row) and humans (final row). The apes show mostly separate sternebrae, but with some partially fused sternebrae, and one with evidence of vertical fusion of the two sternal halves. Middle photographs, upper and lower, show en face sternums with some separate, some partially fused and some completely fused sternebrae in adult (17–30 year old) cretins. These can be compared to images of saggital sections of sternums with completely separate sternebrae in immature cretins in reference [42]. Right side, three photographs show increased fusion of sternebrae in cretins including one cretin in which the sternum is entirely like a normal human, and one cretin showing a vertical line of fusion (perhaps from the original two halves of the developing sternum) in three cretins aged 67–80 years. En face photographs from Basle specimens, 64, 65, 66, 84 and 85. (0.09 MB DOC) [file pone.0013018.s007.doc]

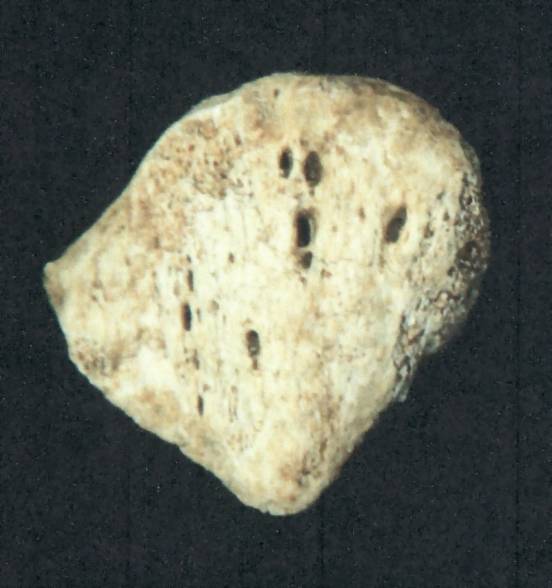

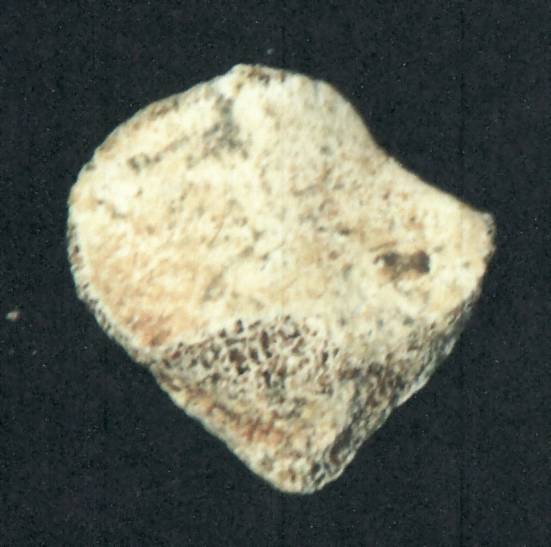

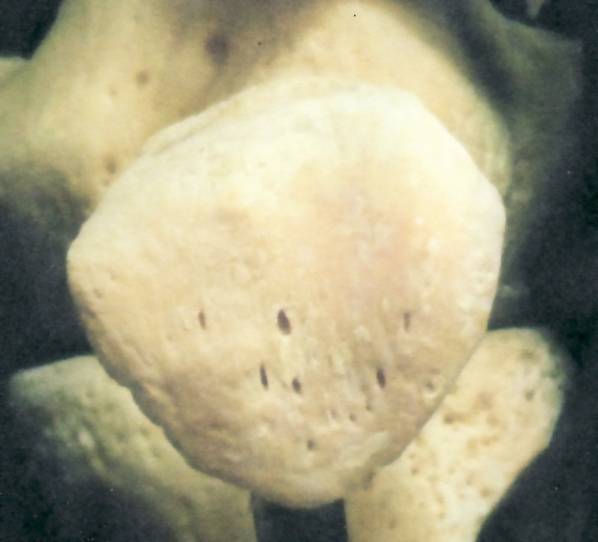

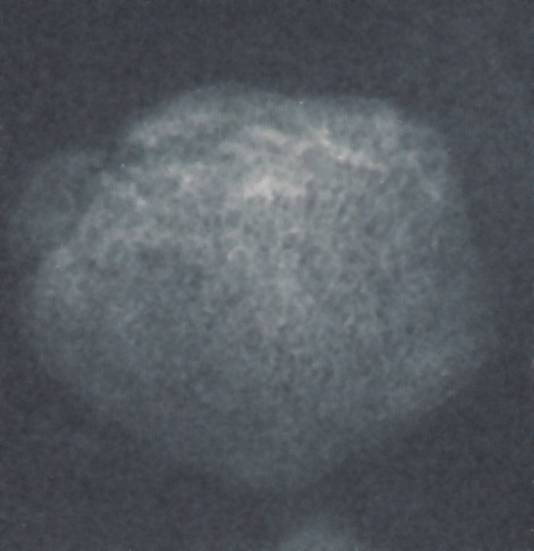

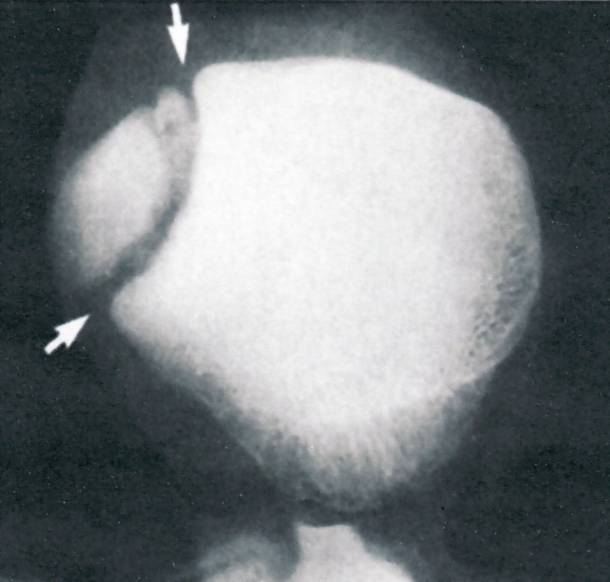


**a**

**b**

**e**

**c**


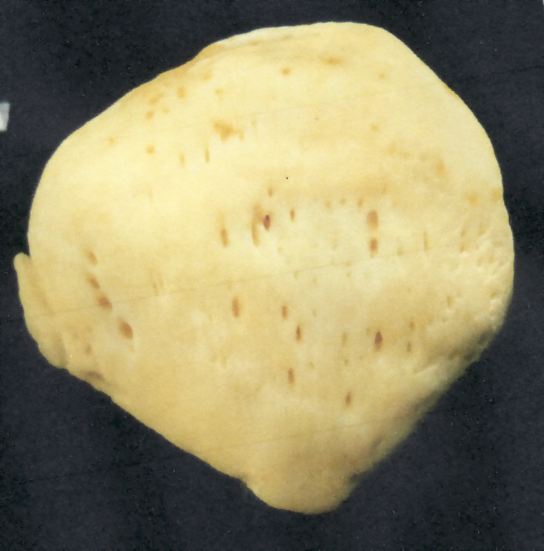


**f**

**d**

Supplement: Figure S7 — Patella of LB1 compared to developing unaffected humans and young adult cretin. The right patella of LB1 is shown in anterior view (a) and posterior view (d). Radiographic views of right patellae of a 15 year old (b) and of 8 year old humans (e). Anterior views of right patellae of 28 year old cretin, Basle specimen 66 (c) and 40 year old cretin, Basle specimen 578, f). Short white arrows show limits of concave facets for articulation of epiphyses (when present); red arrows show line of fusion of epiphyses when present. (0.73 MB DOC) [file pone.0013018.s008.doc]

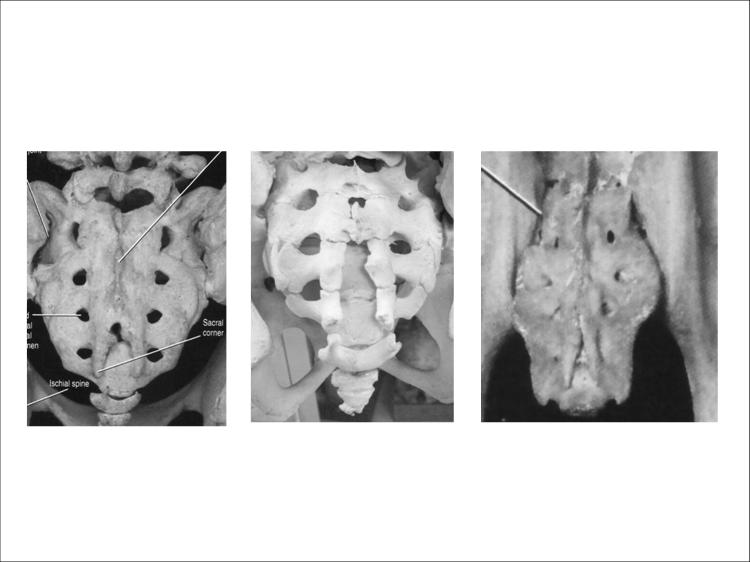

Supplement: Figure S8 — Sacra of hypothyroid cretin compared to unaffected human and chimpanzee. Non-fusion of the many parts of the cretin sacrum (middle) is clear. Normal human (left) and chimpanzee (right) show the fused adult state. (0.08 MB DOC) [file pone.0013018.s009.doc]
